# Isolation and expansion of pure and functional γδ T cells

**DOI:** 10.3389/fimmu.2024.1336870

**Published:** 2024-02-15

**Authors:** Tamara Verkerk, Anouk T. Pappot, Tineke Jorritsma, Lisa A. King, Mariël C. Duurland, Robbert M. Spaapen, S. Marieke van Ham

**Affiliations:** ^1^ Department of Immunopathology, Sanquin Research, Amsterdam, Netherlands; ^2^ Landsteiner Laboratory, Amsterdam UMC, University of Amsterdam, Amsterdam, Netherlands; ^3^ Amsterdam Institute for Infection and Immunity, Amsterdam, Netherlands; ^4^ Department of Medical Oncology, Amsterdam UMC, Vrije Universiteit Amsterdam, Amsterdam, Netherlands; ^5^ Cancer Center Amsterdam, Amsterdam, Netherlands; ^6^ Swammerdam Institute for Life Sciences, University of Amsterdam, Amsterdam, Netherlands

**Keywords:** gamma delta (γδ) T cells, isolation and expansion, functional validation, method comparison, high purity

## Abstract

γδ T cells are important components of the immune system due to their ability to elicit a fast and strong response against infected and transformed cells. Because they can specifically and effectively kill target cells in an MHC independent fashion, there is great interest to utilize these cells in anti-tumor therapies where antigen presentation may be hampered. Since only a small fraction of T cells in the blood or tumor tissue are γδ T cells, they require extensive expansion to allow for fundamental, preclinical and *ex vivo* research. Although expansion protocols can be successful, most are based on depletion of other cell types rather than γδ T cell specific isolation, resulting in unpredictable purity of the isolated fraction. Moreover, the primary focus only lies with expansion of Vδ2^+^ T cells, while Vδ1^+^ T cells likewise have anti-tumor potential. Here, we investigated whether γδ T cells directly isolated from blood could be efficiently expanded while maintaining function. γδ T cell subsets were isolated using MACS separation, followed by FACS sorting, yielding >99% pure γδ T cells. Isolated Vδ1^+^ and Vδ2^+^ T cells could effectively expand immediately after isolation or upon freeze/thawing and reached expansion ratios between 200 to 2000-fold starting from varying numbers using cytokine supported feeder stimulations. MACS/FACS isolated and PHA stimulated γδ T cells expanded as good as immobilized antibody mediated stimulated cells in PBMCs, but delivered purer cells. After expansion, potential effector functions of γδ T cells were demonstrated by IFN-γ, TNF-α and granzyme B production upon PMA/ionomycin stimulation and effective killing capacity of multiple tumor cell lines was confirmed in killing assays. In conclusion, pure γδ T cells can productively be expanded while maintaining their anti-tumor effector functions against tumor cells. Moreover, γδ T cells could be expanded from low starting numbers suggesting that this protocol may even allow for expansion of cells extracted from tumor biopsies.

## Introduction

γδ T cells are considered to be potent cells to combat infections and malignancies because of their strategic localization in mucosal tissues, specific recognition of target cells and direct response upon activation. Like αβ T cells, γδ T cells have a potent capacity to elicit a cytotoxic response upon specific activation via release of TNF-α, IFN-γ, perforins and granzymes (1–3). Unlike αβ TCRs, γδ-TCR triggering is typically independent of antigens presented on major histocompatibility complex I or II (MHC-I/II). In addition, γδ T cells are fully mature when they leave the thymus, have a broad range of antigen recognition and can consequently respond rapidly to antigen encounter. Similar to both αβ T cells and natural killer (NK) cells, γδ T cells express NKG2D which can detect stress-induced signals on tumors and infected cells (4). In addition, these cells also share other receptors with innate NK cells such as FcγRIIIa (CD16) which is required to recognize opsonized tumor cells, DNAX accessory molecule (DNAM-1, CD226) and natural cytotoxicity receptors (NCRs) NKp46, NKp44 and NKp30 which promote anti-tumor cytotoxicity (5, 6).

γδ T cells can be divided into different subsets based on the composition of the TCR chains of which Vδ1 and Vδ2 are two major γδ T cells subsets found in humans. Both types can be found in peripheral blood of which the majority are Vδ2 T cells. Vδ1 T cells mainly reside in tissues and mucosa, such as the skin, spleen, gut, and lungs (7–10).

The majority of γδ T cells are activated in an MHC-I independent manner and only a few γδ T cell clones have been reported to become activated by classical MHC-I molecules (11–13). Vδ1 T cells recognize MICA/B and ULBPs on cells through γδ TCR and NKG2D engagement or CD1c/d on antigen presenting cells (1, 14). Vδ2 T cells harbor a TCR that can be activated through surface expression of the butyrophilin (BTN) BTN3A1/BTN2A1 complex, modulated by pathogenic changes in cells (15, 16). Accumulation of intracellular phosphoantigens (pAgs) instigates a conformational change of the BTN complex, via pAgs binding to the intracellular BTN3A1 B30.2 domain, which is important for Vδ2 T cell activation (15, 17).

The mode of action of γδ T cells in an anti-tumor response comprises direct cytolytic activity via NKG2D, FcyRIII, Fas/Fas-ligand, DNAM-1 receptor-ligand interaction and the TNF related apoptosis inducing ligand (TRAIL) pathway resulting in production of perforins, granzymes, IFN-γ and TNF-α (4, 5, 18–23). In concurrence, γδ T cells can also activate other immune cells (24, 25), for instance activate NK cells via co-stimulation, promote dendritic cell (DC) maturation and support initiation of a humoral immune response (26–31).

Interestingly, Vδ1 and Vδ2 T cell subsets can represent up to 50% of CD3^+^ tumor infiltrating lymphocytes (TILs), although this varies between tumor type and stage (1, 32–36). Recently, Knight and colleagues found increased frequencies of both Vδ1 and Vδ2 T cells in patients with chronic myeloid leukemia (CML) compared to age-matched healthy donors and demonstrated primary CML specific lysis by autologous Vδ1 T cells (35). Some studies found correlations between tumor infiltrating γδ T cells with a favorable clinical outcome. For example, the study conducted by Gentles and co-authors found that intra-tumoral signatures of γδ T cells were the most significant, favorable anti-tumor prognostic factor by analyzing leukocyte frequencies of 22 cell types from 25 pooled human cancers (37). It remains to be established though if tumor infiltrating γδ T cells can be used as a prognostic factor (1, 25, 33, 38, 39). Motivation for the application of γδ T cells in immunotherapy is driven by the distinctive MHC independent activation, meaning that its application is not restricted to an autologous setting. In addition, they have a potential to be used for tumors showing immune escape through downmodulation of MHC expression. Immunotherapy based on γδ T cells involves *in vivo* stimulation of Vδ2 T cells through intravenous administration of pamidronate or zoledronate, which are bisphosphonates stimulating the conformational change of the BTN3A1/BTN2A1 complex, and autologous or allogenic reinfusion of *ex vivo* zoledronate driven expanded γδ T cells (3, 15, 40–42). These treatments have been shown to have minor adverse effects and to be effective for a number of patients suffering from for example, pancreatic, lung, liver and hematological cancers, especially in combination with other conventional therapies (3, 40, 43–53).

The low number of γδ T cells in peripheral blood (0.5-5% of T cells) and, especially, in tumor tissue represents a major challenge for their application in fundamental, preclinical and *ex vivo* studies (54–56). Therefore, expansion of γδ T cells is often applied to study these cells.

Scientific reports in which expanded γδ T cells are used are mainly subset specific and vary in isolation and expansion strategies. Most used methods are based on the use of bisphosphonates zoledronate, pamidronate or bromohydrin pyrophosphate (BrHPP) to activate Vδ2 T cells specifically residing in total PBMCs (49, 57–59). These compounds inhibit the IPP processing enzyme farnesyl diphosphate synthase (FDPS), consequently increasing IPP levels and subsequent the BTN3A1/BTN2A1 structure (15). Zoledronate based expansion of Vδ2 T cells in total PBMCs can generate over 90% pure Vδ2 T cells, but shows strong variation in yields between donors (59–63). Other methods focus on TCR activation with, for instance, lectins such as phytohaemagglutinin (PHA) or anti-CD3 (OKT3) with or without anti-CD28 (64). In literature, TCR targeting activation methods are generally used to produce large numbers of Vδ1 T cells (65, 66). Other, less conventional methods have been described. For instance, Siegers and colleagues showed activation with the mitogen Concanavalin A can expand presorted Vδ1 and Vδ2 γδ T cells (67). Lastly, K562 cells are used as feeder cells which are modified to express various molecules such as CD40L, CD70, CD80, CD86, CD83, CD137L and artificial membrane bound cytokines like IL-15 (58, 68, 69). Generally, IL-2 is used to promote activation, growth and proliferation, often combined with other cytokines like IL-4, IL-7, IL-15, IL-18 and IL-21 (58). Isolation of γδ T cells usually incorporates an αβ T cell depletion step, occasionally combined with an additional NK cell or undesired γδ subset depletion step, either before or after the expansion period.

The issues that accompany the current described methods is that they are variable, solely applicable to specific subsets and may yield unpredictable or impracticable levels purity of the (final) product. Hence, there is a need for a practical protocol that can be used to specifically isolate Vδ1 and Vδ2 T cells followed by an expansion method that assures purity and maintains their functional properties. Standardized isolation and expansion protocols would allow for comparison between studies and benefit the growth of knowledge on γδ T cell differentiation, function and targets.

Here, we report an isolation and expansion method which reproducibly generates high and >99% pure Vδ1 and Vδ2 T cell numbers after 14 days of expansion using PBMC as starting material. This method focuses first on establishing γδ T cell populations through Vδ1 and Vδ2 T cell specific isolation, which can subsequently be expanded to over a 1000-fold increase. In addition, we show that these γδ T cells can effectively be activated and kill tumor cells. Moreover, this method can be used to expand cells from low starting numbers or frozen, previously expanded cells. Together, this allows investigators to reproducibly generate large batches of γδ T cells to study their biology.

## Materials and methods

### Isolation of γδ T cells from PBMCs

Buffy coats were collected from healthy donors (Sanquin Blood Supply, Amsterdam, the Netherlands) who provided written informed consent for the use of their donation for research. Peripheral blood mononucleated cells (PBMCs) were isolated from buffy coats using Lymphoprep (Axis-Shield PoC AS, Dundee DD2 1XA, Scotland) density gradient. For direct targeted isolation of γδ T cells, PBMCs were incubated with PE conjugated mouse anti-human Vδ1 TCR or APC/PE conjugated mouse anti-human Vδ2 TCR for 30 minutes on ice (**Table 1**). For PAN γδ T cell isolation, unconjugated anti-PANγδ TCR was used (**Table 1**). The PBMCs were washed with PBS/0.1%BSA and incubated with anti-mouse IgG microbeads (Miltenyi) prior to positive MACS isolation according to manufacturer’s protocol. Subsequently, the collected γδ T cells were purified using a FACS sort for PE^+^ (Vδ1) or PE/APC^+^ (Vδ2) cells. For PANγδ isolation, cells were stained with anti-IgG1 (APC) prior to FACS sort (**Table 1**). For untouched, pan γδ T cell isolation, the TCRγ/δ+ T Cell Isolation Kit (Miltenyi) was used according to manufacturer’s protocol. The purity of the isolated γδ T cells was assed prior to cell culture.

**Table 1:** Antibodies.

	Source	Identifier	Dilution
Mouse anti human IFN-γ PE	Biolegend	502509	1:100
Mouse anti human TNF-α Pe-Cy7	BD	557647	1:100
Mouse anti human/mouse granzyme B BV421	Biolegend	396414	1:150
Mouse anti human BTN3A1/2/3	R&D systems, Biotechne	MAB7136	1:100
Mouse anti human PANγδ TCR	Beckman Coulter	IM1349	1:100
Mouse anti human Vδ1 TCR PE	eBioscience	12-5679-42	1:100
Mouse anti human Vδ1 TCR APC	Miltenyi	130-118-968	1:100
Mouse anti human Vδ2 TCR PE	Biolegend	331408	1:200
Mouse anti human Vδ2 TCR APC	Biolegend	331418	1:200
Mouse anti human CD3 BV510	BD	563109	1:200
Mouse anti human CD25 BV605	BD	562661	1:100
Mouse anti human CD27 PE-Cy7	Invitrogen	25-0279-42	1:200
Mouse anti human CD45RA PE	BD	555489	1:200
Mouse anti human CD69 BUV395	BD	564364	1:200
Mouse anti human CD137 BUV661	BD	741642	1:200
Goat anti mouse IgG1 AF633	ThermoFisher Scientific	A-21052	1:200

### Cell culture

The target cell lines WM9 and WiDr (kindly provided by prof. J.J. van der Vliet, Amsterdam UMC, the Netherlands) were cultured in DMEM (Gibco) supplemented with 10% FCS (Serana), antibiotics (PenStrep, Invitrogen), 1% L-glutamine (Gibco) and 0.05 mM BME (Sigma). The target cell line HAP1 and HT29 cell line, and the EBV-LCLs used as feeder cells were cultured in IMDM (Gibco) supplemented with 10% FCS and antibiotics. Freshly isolated, thawed or 14-day cultured γδ T cells were expanded using the protocol as described in this paper. In short, γδ T cells per well were expanded in IMDM containing feeder cells (0.1x10^6^ irradiated EBV-LCLs (50Gy) and 1x10^6^ PBMCs (30Gy) per ml), 5% heat inactivated human serum (HS, Sanquin), 5% FCS and antibiotics supplemented with 120 U/mL IL-2 (Peprotech), 20 ng/mL IL-7 (Research grade, Miltenyi), 20 ng/mL IL-15 (Peprotech) and 1 μg/mL Phytohaemagglutinin (PHA, HA16 Remel™, Thermo Fisher Scientific) at the start of the culture. Cultures with 150 cells started in a 96-well plate in a total volume of 100 µl, 15.000 cells started in a 48-well plate with a total volume of 500 μl and cultures starting at 150.000 cells were performed in 24-well plates with a total volume of 1ml. Cell cultures were harvested, counted and replated in 24-well plates at day 7 and day 10 (15.000 and 150.000 start) or day 17 (150 start) to maintain these cytokine concentrations and a cell density of 0.5 x 10^6^ cells per ml. All cells were cultured at 37°C and 5% CO_2_.

### Immobilized antibody mediated activation

Anti-PANγδ (IMMU510, Beckman Coulter), anti-Vδ1-PE and anti-Vδ2-PE (**Table 1**) were coated on tissue treated 24-well plates at 1 μg/ml in a total volume of 500 μl PBS, overnight at 4°c. The PBS was removed and 1 million PBMCs were added in 1 ml of medium per well: IMDM containing 10% FCS, 5% HS, antibiotics supplemented with 120 U/mL IL-2, 20 ng/mL IL-7, 20 ng/mL IL-15. Cells were provided with additional medium, harvested, counted and replated as described. The cells were cultured at 37°C and 5% CO_2_ for a 14-day period. After expansion, αβ T cells were depleted using a TCRα/β T cell depletion kit (Miltenyi) according to manufacturer’s protocol. The percentage of Vδ1^+^ T cells and Vδ2^+^ T cells in the total PBMCs after isolation and after 14-days expansion was used to determine the absolute number of Vδ1^+^ T cells and Vδ2^+^ T cells. The fold expansion was calculated through division of the absolute number of Vδ1^+^ T cells and Vδ2^+^ T cells after expansion by the absolute starting numbers.

### PMA/Ionomycin stimulation

γδ T cells were stimulated with 100 ng/mL PMA (Sigma-Aldrich) and 1 µg/mL ionomycin (from Streptomyces conglobatus, Sigma-Aldrich) for 1.5 hours at 37°C and 5% CO_2_. Brefeldin A (1:1000, BD) was added to the medium at the start of stimulation for intracellular measurement of IFN-γ, TNF-α and granzyme B. For analysis, the cells were transferred to a V-bottom plate and washed with cold PBS/0.5%BSA prior to staining. Production of IFN-γ, TNF-α and granzyme B was measured using flow cytometry.

### Coculture assays

The target cells WM9, WiDr, HT29 and HAP1, were plated one day prior to the coculture with γδ T cells at a density of 15.000 cells/well in a flat-bottom 96-well plate and treated O/N with 10 µM pamidronate (PAM, Sigma) in complete IMDM or DMEM. After the O/N culture, the wells were carefully washed to remove dead cells and freshly expanded γδ T cells were added at a 10:1 ratio (E:T) in a total volume of 100 μl medium (IMDM). After a 5-hour coculture, the cells were harvested from the plates and transferred to V-bottom plates. Target cells that remained attached after the initial harvest were removed using trypsin and added to the V-bottom plate. The percentage of dead target cells and activation marker expression on γδ T cells were measured using flow cytometry.

### Flow cytometry

Antibodies used are listed in **Table 1**.

PBMCs or γδ T cells were washed with PBS prior to staining. Extracellular staining was performed by incubation with specific antibodies and LIVE/DEAD NEAR-IR (1:1000, Thermo Fisher Scientific) diluted in PBS for 30 min. on ice in the dark. The cells were washed twice with buffer (PBS/0.5% BSA) and either resuspended in PBS and directly analyzed or fixated using the BD Cytofix/Cytoperm fixation and permeabilization kit (BD Bioscience) according to the manufacturer’s protocol. For intracellular staining, cells were permeabilized and incubated with antibodies targeting IFN-γ, TNF-α or granzyme B diluted in permeabilization buffer for 30 min. on ice. Subsequently, the cells were washed twice and resuspend in PBS prior to analysis. To asses BTN3A1/2/3 levels on target cells, the cells were incubated with NEAR-IR and anti-BTN3A1/2/3 for 30 min. on ice. Next, the cells were washed twice with PBS and incubated with anti-IgG1 for 30 min on ice, followed by another two wash steps and resuspended in PBS prior to analysis. Stained cells were analyzed or sorted on BD flow cytometers (LSR-II, Fortessa, FACSymphony or ARIA-II) and analyzed using FlowJo™ software version 10.9.0 (Ashland, OR: Becton, Dickinson and Company; 2023).

For analysis of γδ T cells in PBMCs, after isolation and stimulation, the cells were gated on single cells, time, NEAR-IR negative events and CD3, Vδ1 or Vδ2 positive events. For analysis of target cell killing, γδ T cell were distinguished using the gating strategy as described and remaining cells were gated for single cells and time before NEAR-IR evaluation representing dead cells.

### Statistical analysis

Statistical testing was done by a student’s T-test or a one-way ANOVA followed by a Tukey’s multiple comparison test and are indicated in the figure legends. The statistical analysis was performed using GraphPad Prism version 10.0 for Mac OS (GraphPad Software, Boston, Massachusetts USA). Differences were considered significant when p≤0.05.

## Results

### Vδ2 T cells directly isolated from PBMCs can reach a 734-fold expansion on average during a 14-day culture period

**Figure 1:**
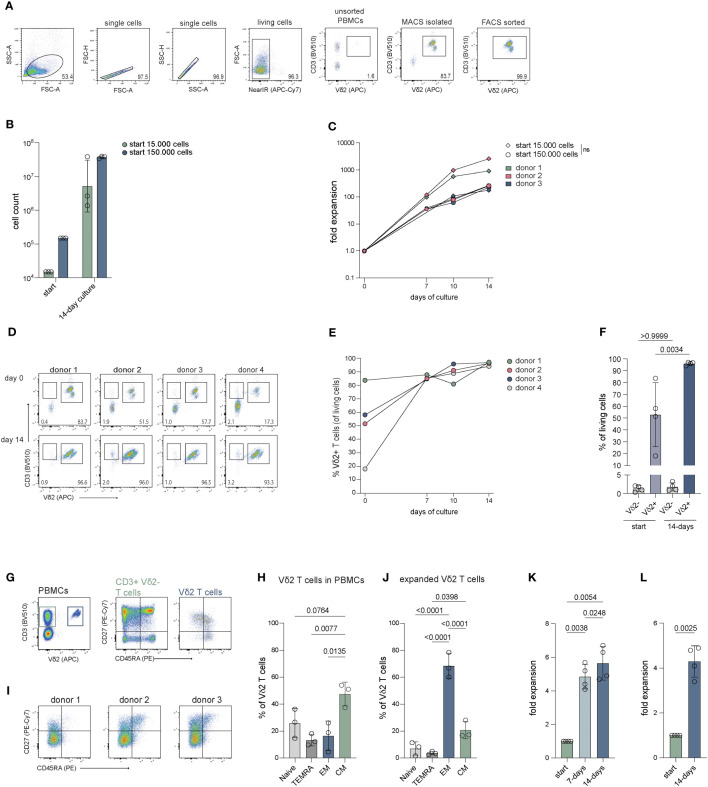
Cell count and fold expansion of Vδ2^+^ T cells after 14 days of expansion. The cells were isolated through mouse anti-human Vδ2 TCR and anti-mouse IgG bead MACS enrichment, with or without additional FACS sort, and expanded straight after isolation or previously expanded Vδ2^+^ T cells were submitted to a new round of expansion. Expanded cells were stained for CD27 and CD45RA to determine their differentiation status. **(A)** Representative flow cytometry plots and gating strategy showing the percentage of Vδ2^+^ T cells in unsorted PBMCs, after Vδ2 TCR specific MACS isolation and after an additional FACS sort for Vδ2^+^ T cells. **(B)** The number of Vδ2^+^ T cells used at the start of expansion, 15.000 and 150.000, and the number of cells yielded after the 14-day expansion (*n=3*). **(C)** The fold expansion of the Vδ2^+^ T cells for each donor of day 7, 10 and 14 relative to the start numbers, 15.000 and 150.0000, respectively (*n=3*). **(D)** Flow cytometry plots showing the percentage of the Vδ2^+^ cells after isolation (top row) using only the anti-Vδ2 TCR antibody combined with anti-mouse IgG beads and MACS separation, without FACS sort, and after a 14-day expansion period (bottom row) for four donors. **(E)** The percentage of Vδ2^+^ T cells after 7, 10 and 14 days of expansion and **(F)** the percentage of CD3^+^Vδ2^-^ T cells and CD3^+^Vδ2^+^ T cells before and after 14 days of expansion. **(G)** Representative flow cytometry plots showing the frequency of CD3^+^Vδ2^-^ and CD3^+^Vδ2^+^ T cells in PMBCs (*left plot*) and the frequency of naïve (T_naive_, CD27^+^CD45RA^+^), terminally differentiated Effector Memory RA (T_EMRA_, CD27^-^CD45RA^+^), Effector Memory (T_EM_, CD27^-^CD45RA^-^) and Central Memory (T_CM_, CD27^+^CD45^-^) T cells for the CD3^+^Vδ2^-^ (*middle plot*) and CD3^+^Vδ2^+^ T cells (*right plot*) in PMBCs before isolation. **(H)** Summary of the percentage of T_naive_, T_EMRA_, T_EM_ and T_CM_ subsets in CD3^+^Vδ2^+^ T cells before isolation, as determined in **(H, I)** Flow cytometry plots of three donors showing the distribution of the T_naive_, T_EMRA_, T_EM_ and T_CM_ subsets in the total Vδ2^+^ T cell population after a 14-day expansion culture. Gating is based on freshly isolated CD3^+^ cells in total PMBCs from a reference donor. **(J)** Summary of the data in **(I, K)** The fold expansion of Vδ2^+^ T cells that were expanded for 14 days that were, straight from culture, submitted to a new expansion culture of 14-days (*n=4*). **(L)** Fold expansion of Vδ2^+^ T cells that were previously expanded for 14 days after which they were cryopreserved for at least four weeks, thawed and submitted to a new expansion culture of 14 days (*n=4*). The data is shown as the mean and standard deviation of the donors and data from each donor represents the mean of triplicates. Data was analyzed by a one-way ANOVA followed by Tukey’s multiple comparisons test **(B, C, F, H, J, K)** or a student’s T-test **(L)**.

Vδ2 T cells are the most prevalent γδ subset in the peripheral blood. To examine if Vδ2 T cells can be efficiently isolated directly from PBMCs and subsequently expanded, magnetic isolation was performed using mouse anti-Vδ2 TCR antibodies and anti-mouse IgG beads (MACS), followed by a FACS sort. MACS isolation generally yielded between 80-90% pure Vδ2^+^ T cells (of living cells) which could be improved to >99% with FACS sort (**Figure 1A**; **Supplementary Figure 1A**).

Culture of the cells was initiated at either 15.000 or 150.000 cells per well to determine the effect of cell seeding density on expansion. During expansion, the percentage of Vδ2^+^ T cells remained stable with a >98% purity with a minor reduction to approx. 95% at day (**Figure 1B**; **Supplementary Figures 1A, B**). Cultures which started with 15.000 cells reached cell numbers of 3.3-38.5 million cells at day 14 and cultures starting with 150.000 cells resulted in 33.9-39.5 million cells after 14 days (**Figure 1C**). There was no significant difference in expansion between the different seeding densities (15.000 versus 150.000 cells) (**Figure 1C**). Direct isolation targeting the Vδ2 TCR thus yields pure Vδ2^+^ T cells populations (>99%) which can successfully be expanded.

### FACS-sorted Vδ2 cells expand equally to non FACS-sorted cells

To assess if the process of FACS sorting following MACS enrichment affects the expansion potential of the isolated γδ T cells, the expansion capacities of isolated Vδ2^+^ T cells with or without FACS sort were compared from the same donors. The expansion of cells that were isolated using only MACS enrichment generated 12-43 million cells (**Supplementary Figure 1C**, adjusted to percentage Vδ2^+^ cells, *n=4*) and displayed a similar fold expansion compared to cells from the same donor that were isolated through both MACS and FACS sort (**Supplementary Figure 1D**, *n=3*). Thus, FACS sorting does not compromise the expansion capacity of Vδ2^+^ T cells.

### The expansion of Vδ2 T cells is not compromised by outgrowth by other cell types

Given the generic character of the feeder-based expansion protocol, other T cell subsets may potentially overgrow the γδ T cells during the expansion phase. To investigate whether the purity of the Vδ2^+^ T cells could be compromised by the contamination of αβ T cells or other cell types, less pure Vδ2^+^ T cells isolated using direct targeting MACS isolation (*n=4*) or pan γδ T cells isolated using untouched MACS isolation (*n=2*) without FACS sort were expanded and significant co-expansion of other cells was monitored.

After direct Vδ2^+^ targeting MACS isolation, the percentage of CD3^+^Vδ2^+^ T cells ranged between 17-84% (**Figure 1D** *top panel*, **E**, **F**) and between 0.4-2% of the isolated cells was CD3^+^Vδ2^-^. Most Vδ2 negative cells that remain after MACS isolation were not T cells (CD3^-^). The percentage of CD3^+^Vδ2^+^ T cells significantly increased to 93-97% after a 14-day expansion period (**Figure 1E**). The CD3^+^Vδ2^-^ cell population expanded alongside the CD3^+^Vδ2^+^ T cells during the 14-day culture though their relative presence did not significantly increase (**Figures 1D, F**). The CD3^-^ cells were no longer present after the expansion (**Figure 1D**, *bottom panel*).

From two donors, γδ T cells were isolated from PBMCs using the TCRγ/δ+ T Cell Isolation Kit (Miltenyi), which comprised of 49% and 80% Vδ2^+^ T cells. Expansion of these γδ T cells yielded 20-27 million cells of which 76-96% were Vδ2^+^ T cells and 3-20% are CD3^+^Vδ2^-^ cells (**Supplementary Figures 1E–G**).

Altogether, this indicates that - during the feeder-based expansion protocol - the purity of Vδ2^+^ T cells is not compromised by overgrowth by other cells, such as αβ T cells.

### The Vδ2 T cells show an effector phenotype after expansion

To assess the phenotype and functionality of the expanded Vδ2^+^ T cells, the cells were immunophenotyped before and after expansion using surface expression of CD27 and CD45RA.

Before expansion, 38-54% of the Vδ2^+^ T cells were primarily of a central memory (T_CM_; CD27^+^CD45RA^-^) phenotype followed by a naïve phenotype (T_naive_; CD27^+^CD45RA^+^), with 15-36% an effector memory phenotype (T_EM_; CD27^-^CD45RA^-^) with 5-26% (**Figures 1G, H**). The percentage of terminally differentiated effector memory RA (T_EMRA_; CD27^-^CD45RA^+^) cells was 9-18%. After expansion, most Vδ2^+^ T cells exhibited a T_EM_ phenotype which comprised 60-79% of the cells, followed by a T_CM_ phenotype containing 17-28% of the cells (**Figures 1I, J**). The T_naive_ and the T_EMRA_ subsets contained the fewest cells with 2-11% and 3-5%, respectively.

### Previously expanded Vδ2 T cells can be expanded further using this PHA based TCR triggering culture method

To examine if Vδ2^+^ T cells can be expanded further after initial expansion, Vδ2^+^ T cells were submitted to a new 14-day expansion culture straight from the first 14-day expansion. The Vδ2^+^ T cells (*n=4*) reached a 4.1-5.7-fold expansion after the first seven days and a 4.7 to 6.7-fold expansion after the second full 14-day expansion period (**Figure 1K**).

To investigate whether 14-day expanded Vδ2^+^ T cells can be expanded further after cryopreservation, frozen 14-day expanded Vδ2^+^ T cells were thawed and submitted to a new 14-day expansion. Frozen, previously expanded Vδ2^+^ T cells (*n=4*) showed a 4.3 to 4.9-fold expansion after 14 days (**Figure 1L**).

Although reduced compared to the first expansion, previously expanded Vδ2^+^ T cells can be expanded further using the expansion protocol described in this report.

### Direct targeted MACS isolation combined with FACS sort and this expansion protocol can be used to expand both Vδ1 and Vδ2 subsets

To investigate if the direct isolation protocol can be used to purify and isolate Vδ1^+^ T cells and if their expansion would be equal to that of Vδ2^+^ T cells, both Vδ1^+^ and Vδ2^+^ T cells were isolated from the same PBMC donors and to compare their expansion competence (*n=3*).

Direct positive isolation of Vδ1^+^ and Vδ2^+^ T cells by magnetic beads (MACS) yielded approx. 90% Vδ1^+^ or Vδ2^+^ pure populations, with purity being increased to > 99% purity by additional FACS sort (**Supplementary Figure 1H**). Over the culture period of 14 days, the two subsets expanded within a similar range through the first week (day 7) with an expansion of 29-fold for Vδ1^+^ and 19-fold for Vδ2^+^ T cells on average **(Supplementary Figure 1I**). At day 14, expansion of both subsets was high, with expansion of the Vδ2^+^ subset exceeding that of the Vδ1^+^ subset with an average of 1248-fold over a 380-fold for Vδ1^+^ T cells (**Supplementary Figure 1I**).

This isolation and expansion method can thus be applied to both Vδ1^+^ and Vδ2^+^ T cells to yield high cell numbers for downstream use.

### Specific isolation combined with PHA activation generates more pure cell populations compared to specific stimulation methods

Activation and expansion through specific stimulation of γδ T cells in total PBCMs by γδ TCR targeting, using immobilized antibodies is often used in the field to obtain Vδ1^+^ T cells, Vδ2^+^ T cells or a mixture of γδ T cell populations (70–72). To determine how the efficacy of our specific isolation and expansion protocols of γδ T cells (MACS+FACS) and PHA activation is similar to these strategies, the purity and fold expansion of Vδ1 and Vδ2 T cells obtained through both methods were analyzed. PBMCs were either subjected to anti-PANγδ TCR, anti-Vδ1 TCR or anti-Vδ2 TCR mediated MACS isolation, followed by FACS sort, or PBMCs were plated on antibody coated plates directly. Sorted cells were stimulated with PHA, feeder cells and cytokines. PBMCs were supported with cytokines at the beginning of the 14-day expansion period (*n=4*).

**Figure 2:**
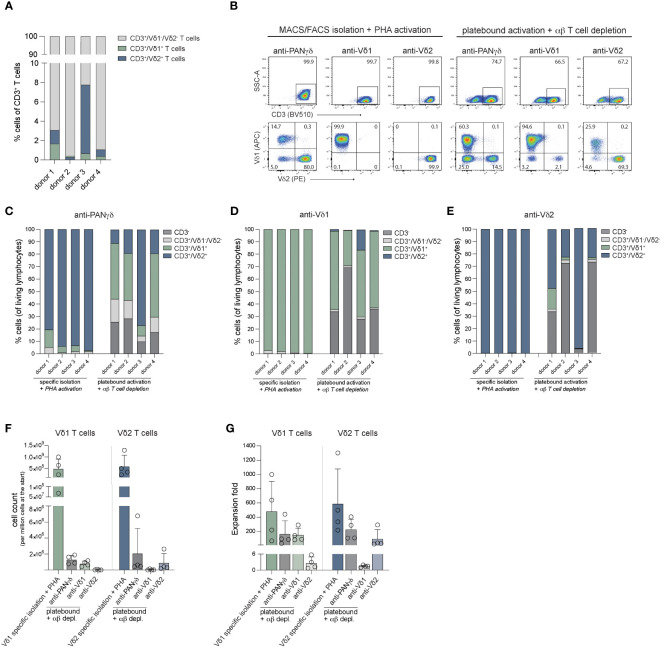
Purity and fold expansion of Vδ1^+^ and Vδ2^+^ cells isolated through MACS/FACS sort using anti-PANγδ/Vδ1/Vδ2 TCR antibodies and activated with PHA or expanded from PBMCs using immobilized anti-PANγδ/Vδ1/Vδ2 TCR antibodies depleted for αβ T cells after expansion. **(A)** The percentage of Vδ1^+^ T cells, Vδ2^+^ T cells and Vδ1^-^/Vδ2^-^ T cells of CD3^+^ cells in PBMCs per donor. **(B)** Representative flow cytometry plots of the percentage of CD3+ cells (*top panel*) and Vδ1^+^/Vδ2^+^ T cells (CD3^+^ gated, *bottom panel*) after 14-days expansion of MACS/FACS isolated + PHA activated cells or cells activated using immobilized (plate bound) antibodies depleted for αβ T cells. **(C)** The distribution of CD3^-^, CD3^+^Vδ1^-^/Vδ2^-^, Vδ1^+^ and Vδ2^+^ cells after 14-days expansion of MACS/FACS using an anti-PANγδ TCR antibody + PHA activated cells or cells activated using immobilized (plate bound) anti-PANγδ TCR antibody depleted for αβ T cells. **(D)** The distribution of CD3^-^, CD3^+^Vδ1^-^/Vδ2^-^, Vδ1^+^ and Vδ2^+^ cells after 14-days expansion of MACS/FACS using an anti-Vδ1 TCR antibody + PHA activated cells or cells activated using immobilized (plate bound) anti-Vδ1 TCR antibody depleted for αβ T cells. **(E)** The distribution of CD3^-^, CD3^+^Vδ1^-^/Vδ2^-^, Vδ1^+^ T cells and Vδ2^+^ T cells after 14-days expansion of MACS/FACS using an anti-Vδ2 TCR antibody + PHA activated cells or cells activated using immobilized (plate bound) anti-Vδ2 TCR antibody depleted for αβ T cells. **(F)** The cell counts generated from 14-days expanded of 1 million Vδ1^+^ and Vδ2^+^ T cells isolated through MACS/FACS sort and activated with PHA + feeder cells, or generated from 1 million PBMCs activated using immobilized (plate bound) antibodies. **(G)** Fold expansion of Vδ1^+^ and Vδ2^+^ T cells isolated through MACS/FACS sort and activated with PHA + feeder cells, or from 1 million PBMCs activated using immobilized (plate bound) antibodies.

Prior to isolation or expansion from PBMCs, Vδ1^+^ and Vδ2^+^ T cells constituted only a small fraction of CD3^+^ T cell population (**Figure 2A**). Expansion of the MACS+FACS sorted cells generated >99% pure CD3^+^ T cells (**Figure 2B**). In contrast, plate bound activation of PBMCs yielded between 75-96% CD3^+^ cells, which still consisted of a significant number of conventional αβ T cells (**Supplementary Figures 2A–C**). To enrich this population for γδ T cells, usually αβ T cells are depleted. which in these experiments reduced the CD3^+^ population to 27-90% after αβ T cell depletion (**Figure 2B**; **Supplementary Figures 2A–C**). Expanded cells after initial PANγδ sort consisted primarily of Vδ2^+^ T cells, while specific PANγδ antibody based expansion followed by an αβ T depletion generated a variable mix of Vδ1^+^ T cells and Vδ2^+^ T cells, contaminated with a fraction of Vδ1^-^/Vδ2^-^ T cells (**Figures 2B, C**). The cells sorted directly with the Vδ1 targeting antibody yielded >99% Vδ1^+^ cells (**Figures 2B, D**). In comparison, plate bound activation with this antibody resulted in a cell population with only 28-62% Vδ1^+^ cells (**Figures 2B, D**). Lastly, cells sorted using anti-Vδ2 antibodies established >99% Vδ2^+^ T cells (**Figures 2B, E**) for all donors compared to 23-96% Vδ2^+^ T cells using anti-Vδ2 plate bound activation (**Figures 2B, E**).

After expansion, cells were counted to assess differences in expansion effectivity of the different methods. One million of anti-Vδ1 sorted and PHA expanded cells generated 482 million Vδ1^+^ T cells on average for the four donors used and one million of cells sorted using the Vδ2 targeting antibody resulted in 588 million Vδ2^+^ T cells on average (**Figures 2F, G**).

The methods using antibody mediated activation and outgrowth of one million PBMCs resulted on average in 7 million Vδ1^+^ T cells (227-fold expansion) and 12 million Vδ2^+^ T cells (159-fold expansion) using anti-PANγδ stimulation (**Figures 2F, G**). Anti-Vδ1 stimulation of one million PBMCs yielded 5 million Vδ1^+^ cells (144-fold expansion) and anti-Vδ2 stimulation generated 5 million Vδ2^+^ T cells (96-fold expansion) (**Figures 2F, G**). There was no significant difference between the cell numbers or fold expansion between directly isolated (MACS+FACS sorted) and PHA stimulated cells versus cells generated through plate bound activation by antibodies combined with αβ T cell depletion after the 14-day expansion.

### The Vδ1 and Vδ2 T cells show an effector phenotype after expansion

**Figure 3:**
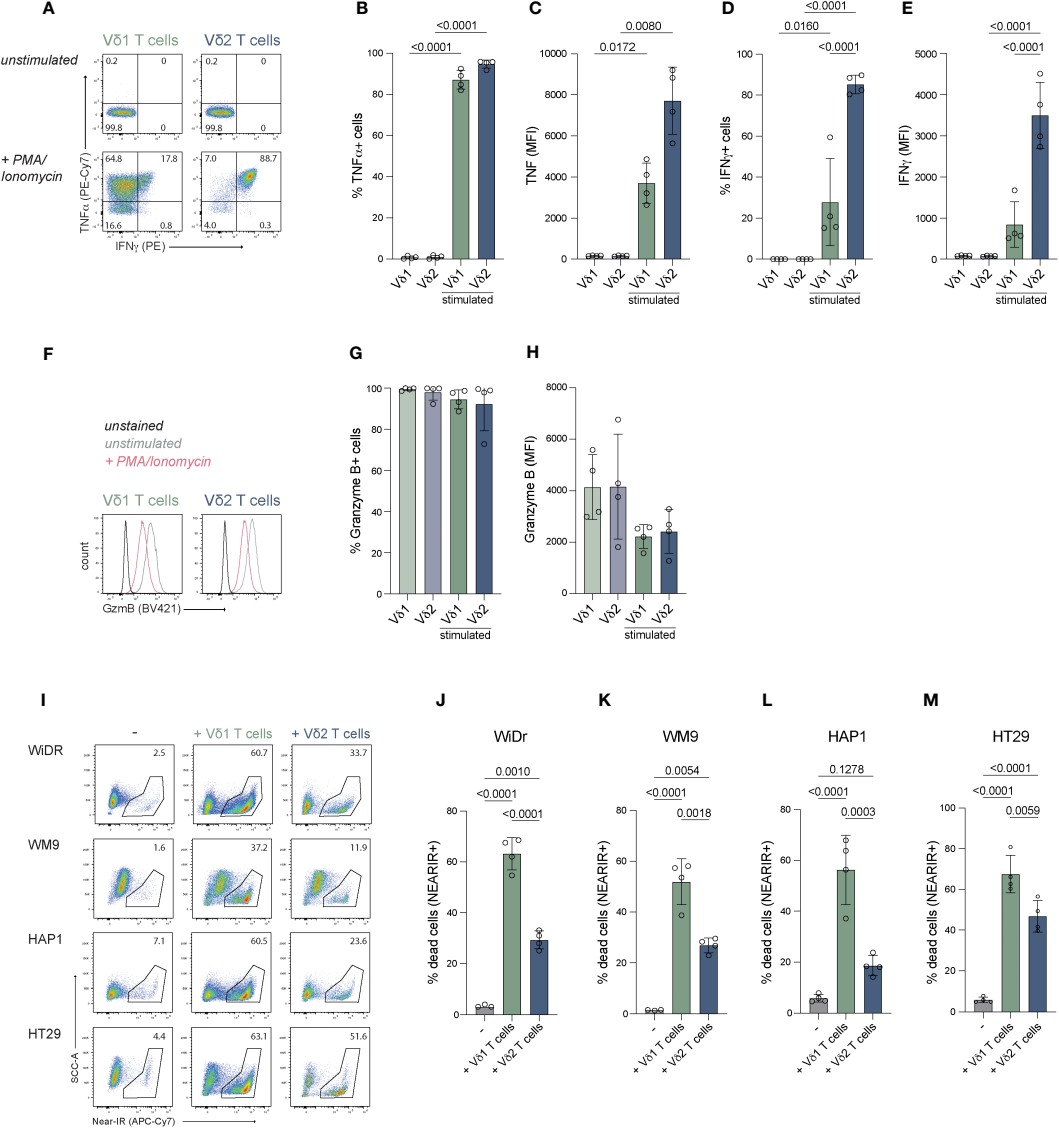
Effector molecule production and anti-tumor function of expanded Vδ1^+^ and Vδ2^+^ T cells. Expanded cells were stimulated with PMA/Ionomycin to assess their potential to produce effector molecules IFN-γ, TNF-α and granzyme **(B)** Tumor killing capacity was assessed with a coculture of freshly expanded γδ T cells with target cells lines WiDr, WM9, HAP1 and HT29. **(A)** Representative flow cytometry plots showing production of IFN-γ and TNF-α by Vδ1^+^ and Vδ2^+^ T cells stimulated with PMA/Ionomycin for 1.5 hour or unstimulated. **(B–E)** Summary of the production of IFN-γ and TNF-α by Vδ1^+^ and Vδ2^+^ T cells stimulated with PMA/Ionomycin for 1.5 hour or unstimulated. The percentage and MFI of the cytokine positive cells is depicted **(F)** Representative flow cytometry plots showing the production of granzyme B by Vδ1^+^ and Vδ2^+^ T cells stimulated with PMA/Ionomycin for 1.5 hour or unstimulated. **(G, H)** Summary of the production of granzyme B by Vδ1^+^ and Vδ2^+^ T cells stimulated with PMA/Ionomycin for 1.5 hour or unstimulated. The percentage and MFI of the granzyme B positive cells is depicted. **(I)** Representative flow cytometry plots showing the frequency of dead (NEAR-IR+) WiDr, WM9, HAP1 and HT29 target cells (*left column*) and target cells cocultured with Vδ1^+^ (*middle column*) or Vδ2^+^ (*right column*) T cells. **(J–M)** Summary of the percentage of dead target cells, WiDr, WM9, HAP1 and HT29, only (-) or with Vδ1^+^ or Vδ2^+^ T cells. Data are shown as the mean and standard deviation of four donors and the data of each donor represents the mean of triplicates. Data were analyzed by a one-way ANOVA followed by Tukey’s multiple comparisons test.

To asses effector potential, expanded Vδ1^+^ and Vδ2^+^ T cells were stimulated with PMA/Ionomycin for 1.5 hours and production of cytokines TNFα and IFNγ, as well as granzyme B release were determined. At least 80% of γδ T cells produced TNF-α (**Figures 3A–C**). Surprisingly, only 14-60% of the Vδ1^+^ T cells produced IFN-γ, while the majority of the Vδ2^+^ T cells were IFN-γ positive (82-90%) (**Figures 3A, D, E**). Production of granzyme B was present in most cells before stimulation (>95%) and cells remained positive upon activation, but showed a significant decrease in MFI for granzyme B staining, possibly indicating early degranulation (**Figures 3F–H**).

These data show that positively isolated Vδ1^+^ and Vδ2^+^ T cells expanded with feeder cells, PHA, IL-2, IL-7 and IL-15 have the capacity to produce IFN-γ, TNF-α and granzyme B.

### The γδ T cells expanded with feeder cells, PHA, IL-2, IL-7 and IL-15 effectively kill target cells

The capacity to induce anti-tumor cytotoxicity by positively isolated and expanded Vδ1^+^ and Vδ2^+^ T cells from four donors was assessed in co-cultures with cell lines derived from different tumor types; WiDr cell line (colon adenocarcinoma), WM9 cell line (melanoma), HT29 cell line (colon carcinoma) and the HAP1 cell line (chronic myelogenous leukemia) which, due to its near-haploidy, is a highly useful cell line for genetic manipulation/gene knock-out approaches in future studies on mechanistic pathways (73).

The Vδ1^+^ T cells derived from all donors showed effective killing of 52-65% WiDr cells, 37-57% WM9 cells, 54-75% of HT29 cells and 31-62% of HAP1 cells (background subtracted) (**Figures 3I–M**). Vδ2^+^ T cells killed significantly fewer of each of these target cells (**Figures 3I–M**). Thus γδ T cells expanded using specific isolation followed by aspecific expansion effectively killed target cells from different origins with superiority of Vδ1^+^ T cells over Vδ2^+^ T cells.

### Pre-treatment of target cells with PAM improves HAP1 cell killing by Vδ2 T cells, but not of WiDr or WM9

Due to dysregulation of the mevalonate pathway, phosphoantigen levels can accumulate in tumor cells resulting in elevated, Vδ2 TCR activating BTN3A1/3A2 complexes on the cell surface. Pre-treatment of cell lines with the aminobiphosphonate pamidronate (PAM) can mimic this increase in phosphoantigen levels and, subsequently, potentially enhance BTN3A1/A2 mediated Vδ2^+^ T cell activation. To test whether including PAM increases activation of the Vδ2^+^ T cells, WiDr, WM9 and HAP1 cells were pretreated with PAM overnight before being subjected to an activation and killing assay.

**Figure 4:**
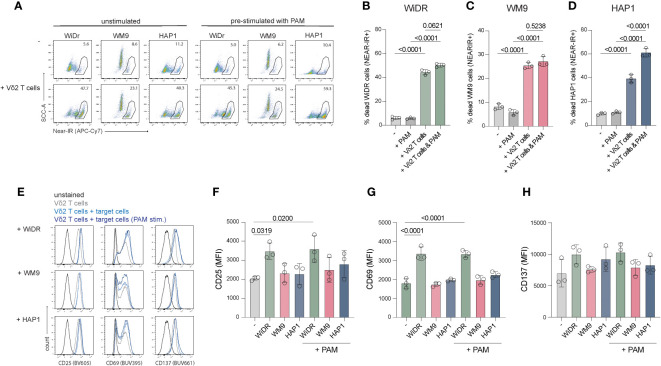
Coculture of freshly expanded Vδ2^+^ T cells with target cell lines WiDr, WM9 and HAP1. 15.000 cells were plated one day prior to the coculture with or without PAM prestimulation. An effector:target ratio of 5:1 was used in a 5-hour coculture. **(A)** Representative flow cytometry plots showing the frequency of dead (Near-IR+) target cells only (*top row*) and target cells cocultured with Vδ2^+^ T cells (*bottom row*), with or without pretreatment with PAM. **(B-D)** Summary of the percentage of dead target cells, WiDr, WM9 and HAP1, only (-) or with PAM treatment and/or addition of Vδ2^+^ T cells. **(E)** Representative histograms plots showing the cell surface expression of CD25, CD69 and CD137 of Vδ2^+^ T cells only or cultured with indicated target cell lines either prestimulated with PAM or not. **(F–H)** Overview of the MFI of CD25, CD69 and CD137 of Vδ2^+^ T cells only (-) or cultured with indicated target cell lines either prestimulated with PAM or not. Gates were set using unstained Vδ2^+^ T cells. Data are shown as the mean and standard deviation of the donors (*n=3*). Data of each donor represents the mean of triplicates. Data were analyzed by a one-way ANOVA followed by Tukey’s multiple comparisons test.

Vδ2^+^ T cells from three different donors effectively killed 35-40% WiDr cells, 15-20% WM9 cells and 25-34% HAP1 cells (background subtracted) (**Figures 4A–D**). Pretreatment with PAM significantly improved HAP1 cell killing by Vδ2^+^ T cells but not of WiDr and WM9 cells (**Figure 4D**). The activation state of expanded Vδ2^+^ T cells via CD25, CD69 and CD137 was also assessed in cocultures with WiDr, WM9 and HAP1 tumor cells (**Figures 4E–H**). Surface expression of CD25 and CD69 was significantly increased on Vδ2^+^ T cells cultured with WiDr cells, indicating that these target cells most strongly activated the Vδ2^+^ T cells (**Figures 4E–G**). To investigate this further, the cell surface levels of butyrophilin of these cell lines were assessed through flow cytometry using an anti-BTN2A1/3A1 antibody. WiDr cells showed a higher cell surface staining compared to WM9 and HAP1 cells, which implies a potential role for butyrophilins in the higher activation of Vδ2 T cells in coculture with WiDr target cells (**Supplementary Figure 3**).

Altogether, these data showed that WiDr cells advance CD25 and CD69 expression on Vδ2^+^ T cells while WM9 and HAP1 cells do not. Pre-stimulation with PAM does not alter expression of activation markers, however, does improve killing of HAP1 cells but not of WiDr or WM9 cells.

### Expansion of Vδ1 and Vδ2 T cells can be achieved from low starting numbers

γδ T cells are of great interest as a means of cellular therapy to treat cancer and understanding the functionality of these cells within tumors is important for the development and optimization of future therapies. However, the absolute amount of γδ T cells isolated from TILs in primary tumors is often very low which complicates further research. Therefore, the applicability of this expansion protocol was evaluated to expand Vδ1^+^ and Vδ2^+^ T cells from very low starting numbers.

**Figure 5:**
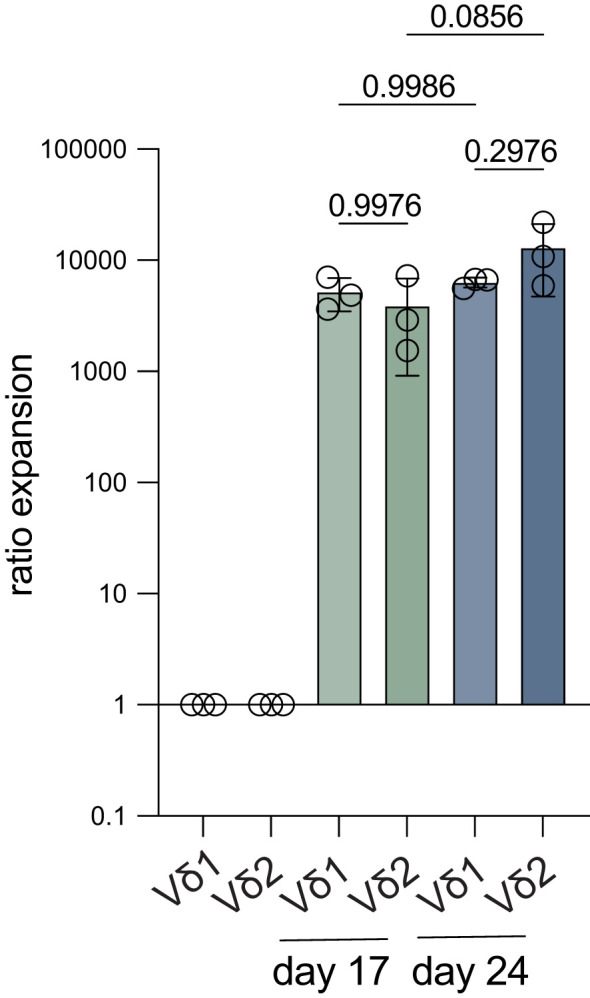
Fold expansion of Vδ1^+^ and Vδ2^+^ T cells starting from low cell numbers. Vδ1^+^ and Vδ2^+^ T cells were isolated from three donors using mouse anti-human Vδ1 or Vδ2 TCR and anti-mouse IgG bead MACS enrichment, followed by FACS sort and 150 cells/well were expanded for 24 days. The fold expansion of Vδ1^+^ and Vδ2^+^ T cells is shown after 17 days and at the end of the expansion culture on day 24 (*n=3*). Ratios were calculated using the cell count on either day 17 or day 24 and compared to the start number (150). Data were analyzed using a one-way ANOVA followed by Tukey’s multiple comparisons test.

Expansion of directly isolated Vδ1^+^ and Vδ2^+^ T cells (purity >95%) from three donors with an initial seeding density of 150 cells per well showed expansion ratios of >1000 fold at day 17 of expansion (**Figure 5**). After a 24-day culture period, the cells had reached a plateau in expansion. There was no significant difference observed between the Vδ1^+^ and Vδ2^+^ T cells.

Thus, this isolation and expansion method is also effective with low amounts of starting material and might potentially be successful in expanding tumor tissue-derived γδ T cells.

## Discussion

We described a methodology to directly isolate γδ T cells from PBMCs using MACS enrichment followed by FACS-based sorting, generating >99% pure γδ T cells. These cells effectively expand and produce effector molecules IFN-γ, TNF-α and granzyme B upon stimulation. In addition, the expanded γδ T cells successfully kill cell lines of different tumor types. More importantly, we show that this method can be applied to expand γδ T cells from low cell numbers (i.e., 150 cells) and may therefore provide a tool to study γδ T cells *ex vivo*.

The here described expansion protocol is based on directly isolating γδ T cells from freshly isolated PBMCs in contrast to most reported expansion protocols which generally deplete αβ T cells and/or CD56 positive cells (64, 68, 74–77). A disadvantage of those methods is that also a fraction of γδ T cells can be removed during this depletion step since they can express CD4, CD8 and CD56 (9, 78–82). On the other hand, the efficiency of depleting other immune cell subsets is often not reported and, in contrast to FACS sort, this depletion is not fully sufficient to remove non-γδ T cells (64). As a consequence, αβ T cells, NK cells and others such as B cells and monocytes might contaminate the γδ T cell cultures (68), which is not preferable because their contribution can result in misrepresentation of the functionality of γδ T cell in *in vitro* assays.

Many established protocols use plate bound antibody activation of γδ T cells as a means of specific expansion (71, 72). Therefore, this optimized protocol was compared with a method using PBMCs stimulated with immobilized γδ T cell specific antibodies followed by an αβ depletion step post-expansion. Pre-expansion isolation and PHA stimulated cells performed at least as good as cells from PBMCs activated with immobilized TCR binding antibodies in terms of fold expansion, but, even after αβ depletion, the purity of the γδ T cells obtained with the plate-bound active activation method was lower than that of MACS/FACS sorted cells. The purity of plate bound activated γδ T cells is likely to be improved with an additional CD56^+^ cell depletion step is added, since NK cells likely persist due to stimulation by IL-15 in culture. Another possibility is selection for the required subset part-way or at the end of the expansion, for example by using the PANγδ TCR isolation kit offered by Miltenyi or TCR targeting isolation. Still, these would involve another time and money consuming step and does not ensure pure populations. For example, we observed that directly after isolation a vast proportion of CD3^-^ cells remains. Again, a steady >99% purity is guaranteed with γδ specific MACS/FACS sort prior to expansion in contrast to other methods showing donor dependent γδ T cell frequencies.

For this experiment a starting number of 6 million PBMCs per donor was used for the plate-bound method and higher numbers the γδ T cells could be achieved by scaling up the starting number. This would however require more working hours and more consumables compared to the pre-isolation method.

It is not completely clear whether having αβ T cells present during the expansion period itself is a benefit or a harm for the expansion of γδ T cells since these cells thrive on the cytokines included in culture. Though, the data on cells isolated through MACS bead separation without FACS sort presented here showed that a small fraction of non-Vδ2 T cells do not overtake γδ T cells during expansion which indicates that the presence of other T cells does not result in an adverse effect on γδ T cell expansion. In addition, the data shown in this report supports that other cell types such as NK cells, B cells and monocytes most likely do not grow out using this MACS isolation and expansion method.

During cultures including other cell types, the γδ T cells may receive survival signals from these cell types that facilitate the expansion. One study suggests that γδ T cells expand better when the other T cells are in the same culture (83). The culture method proposed in this report includes feeder cells comprised of irradiated PBMCs and EBV transformed B cells which may account for these signals. Evaluation of this culture method with addition or substitution of specific (feeder) cells in future may provide more insights into benefit of the other cell types during expansion.

This study is not the first to include a FACS sort for γδ T cell isolation. The added benefit of the protocol described here lies in the combination of MACS isolation and FACS sort, which makes it a very efficient isolation method in terms of yield and consumption of time. Cho and colleagues uses positive selection of Vδ2 T cells solely through FACS sort prior to expansion using K562 feeder cells, anti-CD3/28 and IL-2 (84). Merely a FACS sort is likely to obtain a pure γδ T cell population from PBMCs, however, FACS sorting γδ T cells (0.5-5% of CD3^+^ T cells) is very time-consuming. The pre-enrichment of γδ T cells by MACS isolation (purity γδ T cells of total cells: 80-90%) as described here, greatly reduces the time necessary to FACS sort the cells.

The expansion ratio for Vδ1 and Vδ2 T cells using the protocol described here (starting at 150.000 cells) range between 200 to 2000-fold. These cells can be expanded for a second time, either from culture or cryopreservation, allowing the generation of more cells and repeat or scale up experiments without the need of new γδ T cell isolation. A large fraction of γδ T cells die during or shortly after thawing of the cells after cryopreservation which can be problematic when these cells would immediately be used for functional assays. Restimulating the cells ensures a higher percentage of living cells after culture which can be used for functional analyses. Validation of the phenotype and functionality of the generated γδ T cells may be applied to ensure that the generated cells are still suitable for experimental use.

Ferry and colleagues depleted αβ T cells and CD56^+^ NK cells from PBMCs and stimulated γδ T cell expansion using OKT-3 (anti-CD3) in combination with IL-15 after which they depleted Vδ2 T cells and focused on generating multi-applicable Vδ1 T cells (64). Interestingly, when they compared expansion of the Vδ1 T cells with OKT-3 + IL-15 to PHA + IL-2 or IL-7 they observed a better yield with OKT-3 compared to PHA conditions. Moreover, the OKT-3 activated Vδ1 T cells showed higher expression of activation markers CD69 and NKG2D. However, expansion factors reached with these protocols ranged between 10 to 48-fold using OKT-3 + IL-15 within 20-30 days, while we reached a >400-fold expansion for Vδ1 T cells with PHA and feeder cells in 14 days. This difference could be explained by the use of feeder cells in this protocol. Another reason could be the source of the PHA. The PHA (HA-16) used in this report has repeatedly been confirmed to have high efficiency and the authors potentially used PHA from a different source (73, 85, 86).

We were able to expanded 150 Vδ1 and Vδ2 T cells to 0.8-3.3 million cells in 3.5 weeks, corresponding to an expansion ratio between 5.600-22.000. De Vries and colleagues FACS sorted between 168 and 3775 γδ T cells derived from colon cancer tissue which were expanded for 3-4 weeks using PHA, IL-2 and IL-15, reaching a 2000 to 170.000-fold expansion (87). An explanation for the higher expansion ratios achieved in that study may be a difference in activation state of the isolated cells γδ T cells from tumor tissue. Therefore, exploring the potential of this optimized protocol with γδ T cells extracted from tumor biopsies would be interesting. Another explanation is the IL-2 concentration of 1000 units/ml used while the expansion medium used here contained 120 units/ml IL-2. Whether the protocol described here can be further optimized in terms of cytokine concentrations or inclusion of other immune cell activating cytokines such as IL-4, IL-18 and IL-21, used in several other expansion protocols, needs further investigation.

Functionality of expanded γδ T cells was confirmed by lysis of WiDr, WM9, HT29 and HAP1 tumor cells. To gain more insight in the function of the cells, additional parameters including proliferation, functional persistence and exhaustion marker expression could be investigated.

Interestingly, Correia and colleagues showed higher cytotoxicity towards leukemia-derived cell lines by PHA-expanded γδ T cells over bromohydrin pyrophosphate (HMB-PP)-expanded γδ T (65). Expansion with PHA predominantly generated Vδ1 γδ T cells from peripheral blood lymphocytes with higher expression of NCRs, while HMB-PP stimulation generated Vδ2 cells with low NCR expression. These data suggest that TCR triggering via crosslinking by PHA generates a population of γδ T cells with a higher killing capacity compared to phosphoantigen stimulated expansion. More importantly, the NCR expression by Vδ1 T cells may explain why these cells were superior over Vδ2 cells in the coculture assays demonstrated in this report. Validation and comparison of NCR expression by the expanded cells could provide more insights in the difference in functionality between the two types. In this context, it may be worthwhile to note that despite a similar anti-γδ TCR MACS isolation method, PHA stimulation in the experiments described here show robust Vδ2 T cell expansion in PANγδ sorted cells, while the manuscript by Correia and colleagues showed that PHA stimulation preferences Vδ1 outgrowth over Vδ2 T cells, pointing to the need for more in depth research on the topic.

WiDr tumor cells have a higher cell surface expression of BTN3A1 compared to the WM9 and HAP1 cell lines, which could explain the higher killing percentage and significant increase of CD25 and CD69 on the challenged Vδ2 T cells because BTNs are well described stimulants of the Vδ2 TCR. PAM stimulation of HAP1 cells improved killing of these cells by Vδ2 T cells, while it did not for WiDr and WM9 cells. Stimulation with PAM may promote the structural change of the BTN3A1/A2 complex on HAP1 cells, accelerating Vδ2 TCR signaling, while (regulation of) the confirmational change of BTN3A1/A2 on WiDr and WM9 may differ.

Most reported expansion methods only focus on Vδ2 T cells while Vδ1 T cells also have pro- and anti-tumor potential which renders these cells highly relevant to investigate (42, 88). For example, it has been found that the Vδ1 T cells subset is most prevalent in TILs extracted from melanoma (33, 89). Importantly, these cells were reactive towards both allogeneic and autologous melanoma (89). In addition, Vδ1 T cells showed robust cytotoxicity against colorectal cancer (CRC), hepatocellular cancer (HCC) and leukemia *in vitro* (65, 71, 90). In contrary, it has been shown that IL-17 produced by Vδ1 cells recruits myeloid derived suppressor cells and that they suppress αβ T cell function and DC maturation (91, 92). Therefore, it is important to expand both subsets as is possible with the direct MACS and FACS sort isolation described in this study.

The Vδ1 or Vδ2 subsets are most studied in γδ T cells research while Vδ3 T cells are also found in the blood and in tumors (87). Whether this subtype can also be expanded to generate large quantities of cytotoxic γδ T cells using the protocol described here has to be explored. Lastly, generation of CAR γδ T cells for immunotherapy is emerging in the field. Most protocols involve retroviral transduction of the CAR construct after γδ specific activation, followed by the expansion phase (64, 71, 93, 94). Addition of a transduction to the protocol described here is most likely plausible, though compatibility and productivity would require experimental validation.

## Data availability statement

The original contributions presented in the study are included in the article/**Supplementary Material**. Further inquiries can be directed to the corresponding author.

## Ethics statement

The studies involving humans were approved by Sanquin Ethical Advisory Council, Sanquin, Amsterdam. The studies were conducted in accordance with the local legislation and institutional requirements. The participants provided their written informed consent to participate in this study.

## Author contributions

TV: Conceptualization, Formal analysis, Investigation, Methodology, Validation, Visualization, Writing – original draft, Writing – review & editing. AP: Writing – review & editing. TJ: Writing – review & editing. LK: Writing – review & editing. MD: Validation, Investigation, Writing – review & editing. RS: Funding acquisition, Supervision, Writing – review & editing. Sv: Supervision, Writing – review & editing.

## Supplementary material

Supplementary Figure 1 The purity of Vδ2^+^ T cells isolated with mouse anti-human Vδ2 TCR and anti-mouse IgG bead MACS enrichment followed by a Vδ2^+^ FACS sort during the 14-day expansion. **(A)** Flow cytometry plots of three donors showing the percentage of Vδ2^+^ T cells on day 0 (start), 7, 10 and 14. **(B)** Summary of the percentage of the data in **(A)** (*n=3*). Data of the 150.000 start condition is shown. Cells were isolated using mouse anti-human Vδ2 TCR and anti-mouse IgG bead MACS enrichment, with or without further purification using a FACS sort, or using untouched MACS isolation with the TCRγ/δ+ T Cell Isolation kit. CD3^+^Vδ2^+^ and CD3^+^Vδ2^-^ cells expansion were assessed. **(C)** Cell count of Vδ2^+^ cells isolated using anti-Vδ2 MACS bead separation only after 7, 10 and 14 days of culture to expand the cells (*n=4*). Counts are adjusted to the purity of the cells on the indicated days. **(D)** Comparison of the fold expansion of Vδ2^+^ cells isolated using anti-Vδ2 MACS bead separation only or with an additional FACS purification (*n=3*). The cells from the same donors are compared. Counts that are used to calculate the fold expansion are adjusted to the purity of the cells on the indicated days. **(E)** Flow cytometry plots illustrating the percentage of CD3^+^Vδ2^-^ and CD3^+^Vδ2^+^ of cells isolated with the TCRγ/δ+ T Cell Isolation Kit after 14 days expansion (*n=2*). **(F)** Cell count of CD3^+^Vδ2^+^ T cells isolated using the TCRγ/δ+ T Cell Isolation Kit before and after 14-days expansion (*n=2*) **(G)** Percentage of CD3^+^Vδ2^+^ T cells in the total population isolated using the TCRγ/δ+ T Cell Isolation Kit before and after 14-days expansion (*n=2*). Comparison of the expansion competence of Vδ1^+^ T and Vδ2^+^ T cells of the same donors starting at 150.000 cells per well. The cells were isolated through mouse anti-human Vδ1 TCR or Vδ2 TCR and anti-mouse IgG bead MACS enrichment and further purified using FACS sort. **(H)** Representative flow cytometry plots of the percentage of Vδ1^+^ T cells and Vδ2^+^ T cells in PBMCs, after Vδ1^+^ or Vδ2^+^ T cells specific MACS isolation and after an additional SORT of Vδ1^+^ or Vδ2^+^ T cells before expansion. **(I)** The expansion ratio of Vδ1^+^ compared to Vδ2^+^ T cells after seven days and at the end of the expansion culture on day 14. Ratios were calculated using the cell count on either day 7 or day 14 and compared to the start number (150.000). Data was analyzed using a one-way ANOVA followed by Tukey’s multiple comparisons test **(B, I)** followed by Tukey’s multiple comparisons test or by using a student’s T-test **(D)**.

Supplementary Figure 2 The purity of Vδ1 and Vδ2 T cells generated from PBMCs activated with immobilized **(A)** PANγδ TCR, **(B)** Vδ1 TCR and **(C)** Vδ2 TCR targeting antibodies before and after αβ T cell depletion.

Supplementary Figure 3 Cell surface expression profiles of BTN3A1/2/3 by WiDr, WM9 and HAP1 cell lines. Represented is a flow cytometry plot of WiDr (*green*), WM9 (*pink*) and HAP1 (*blue*) cells incubated with anti-BTN3A1/2/3 and anti-IgG1 (APC). Included is a background control of anti-IgG1 only on WiDr cells (*black*) which is representative for the background on WM9 and HAP1 cells.
