# Supplementary figures and images for: Isolation and expansion of pure and functional γδ T cells
Source: Front Immunol. 2024 Feb 15;15:1336870. doi: 10.3389/fimmu.2024.1336870 (PMC10902048; doi:10.3389/fimmu.2024.1336870)

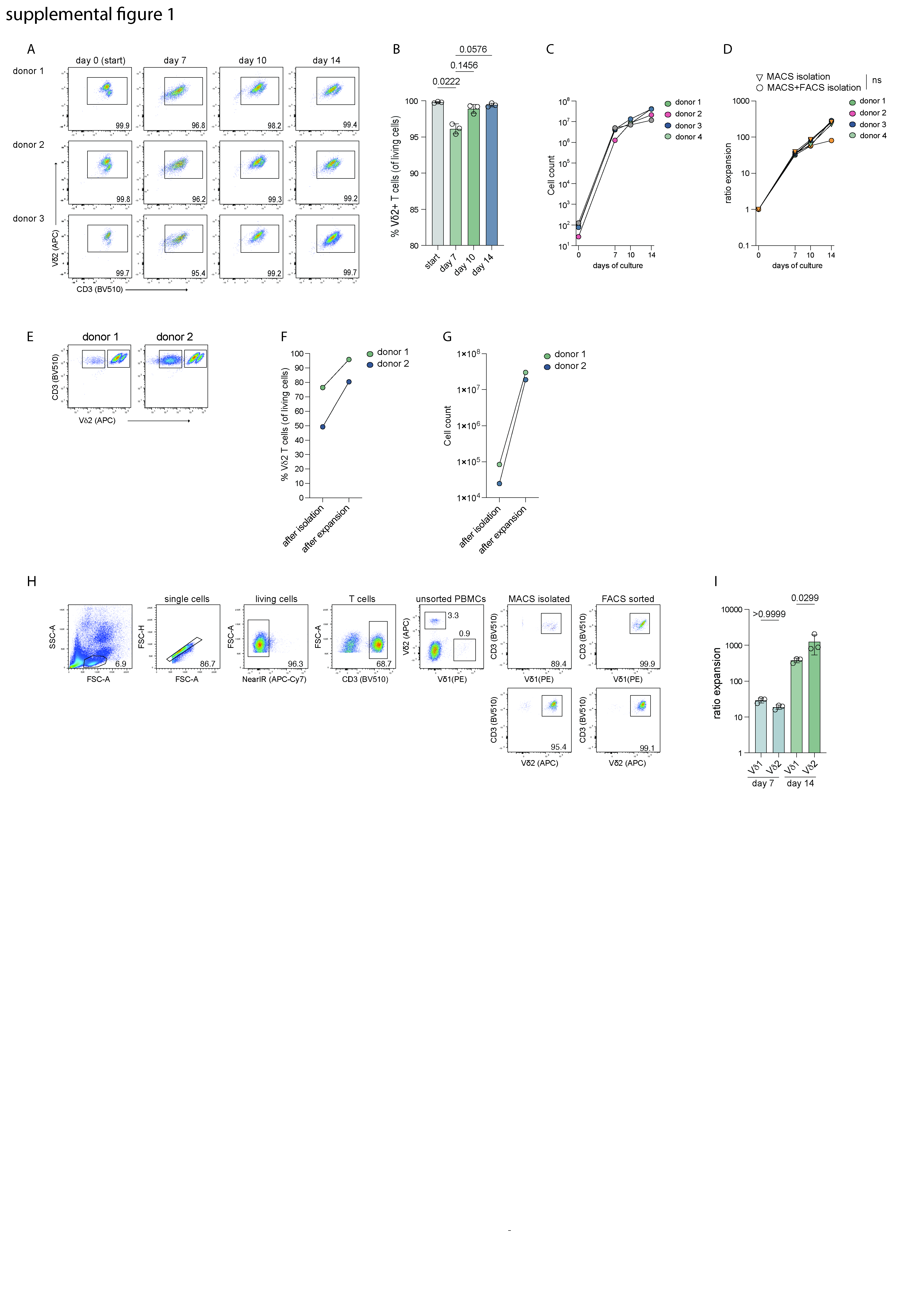

Supplement: Supplementary Figure 1 — The purity of Vδ2+ T cells isolated with mouse anti-human Vδ2 TCR and anti-mouse IgG bead MACS enrichment followed by a Vδ2+ FACS sort during the 14-day expansion. (A) Flow cytometry plots of three donors showing the percentage of Vδ2+ T cells on day 0 (start), 7, 10 and 14. (B) Summary of the percentage of the data in (A) (n=3). Data of the 150.000 start condition is shown. Cells were isolated using mouse anti-human Vδ2 TCR and anti-mouse IgG bead MACS enrichment, with or without further purification using a FACS sort, or using untouched MACS isolation with the TCRγ/δ+ T Cell Isolation kit. CD3+Vδ2+ and CD3+Vδ2- cells expansion were assessed. (C) Cell count of Vδ2+ cells isolated using anti-Vδ2 MACS bead separation only after 7, 10 and 14 days of culture to expand the cells (n=4). Counts are adjusted to the purity of the cells on the indicated days. (D) Comparison of the fold expansion of Vδ2+ cells isolated using anti-Vδ2 MACS bead separation only or with an additional FACS purification (n=3). The cells from the same donors are compared. Counts that are used to calculate the fold expansion are adjusted to the purity of the cells on the indicated days. (E) Flow cytometry plots illustrating the percentage of CD3+Vδ2- and CD3+Vδ2+ of cells isolated with the TCRγ/δ+ T Cell Isolation Kit after 14 days expansion (n=2). (F) Cell count of CD3+Vδ2+ T cells isolated using the TCRγ/δ+ T Cell Isolation Kit before and after 14-days expansion (n=2) (G) Percentage of CD3+Vδ2+ T cells in the total population isolated using the TCRγ/δ+ T Cell Isolation Kit before and after 14-days expansion (n=2). Comparison of the expansion competence of Vδ1+ T and Vδ2+ T cells of the same donors starting at 150.000 cells per well. The cells were isolated through mouse anti-human Vδ1 TCR or Vδ2 TCR and anti-mouse IgG bead MACS enrichment and further purified using FACS sort. (H) Representative flow cytometry plots of the percentage of Vδ1+ T cells and Vδ2+ T cells in PBMCs, after Vδ1+ or Vδ2+ T c [file Image_1.tiff]

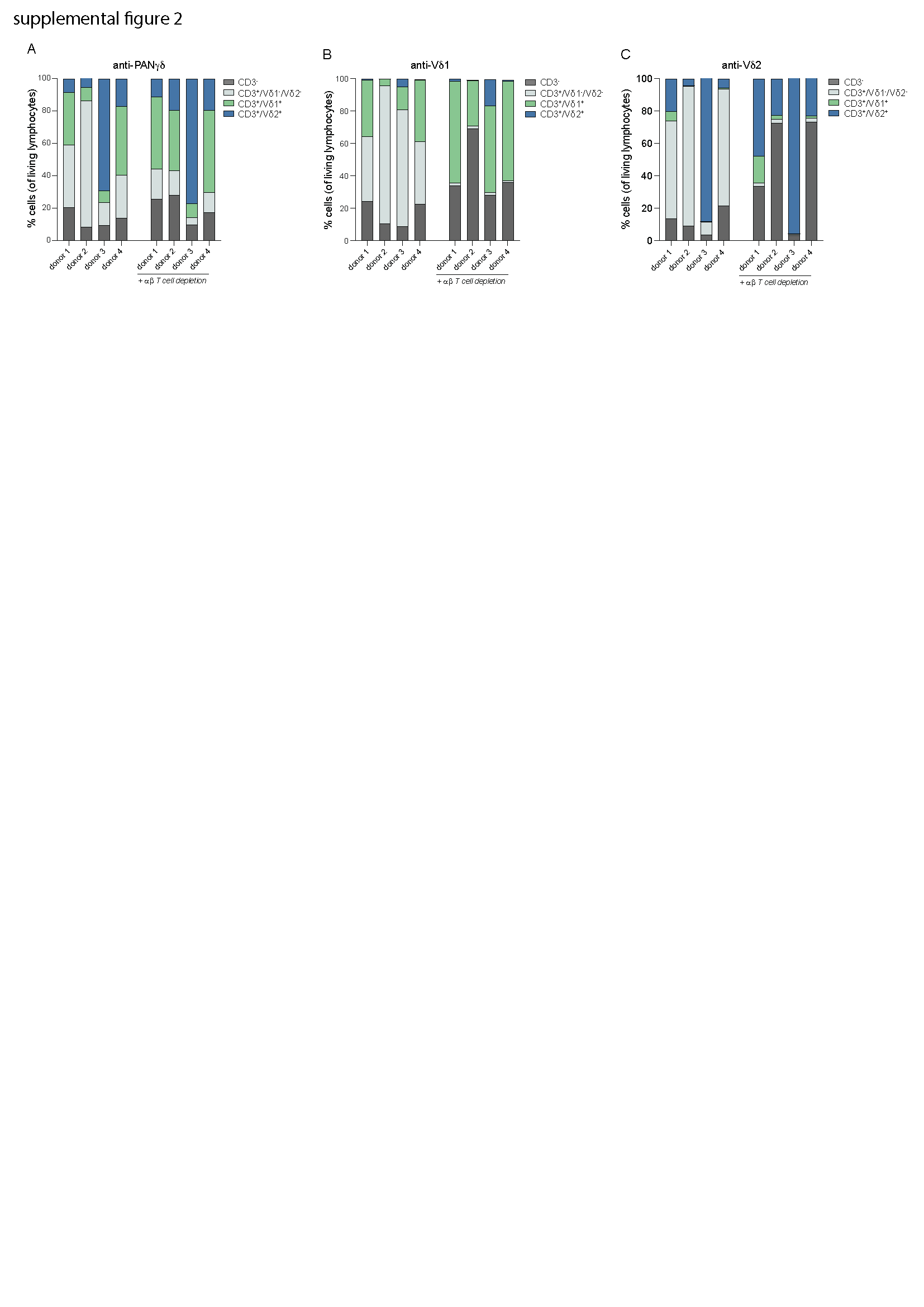

Supplement: Supplementary Figure 2 — The purity of Vδ1 and Vδ2 T cells generated from PBMCs activated with immobilized (A) PANγδ TCR, (B) Vδ1 TCR and (C) Vδ2 TCR targeting antibodies before and after αβ T cell depletion. [file Image_2.tiff]

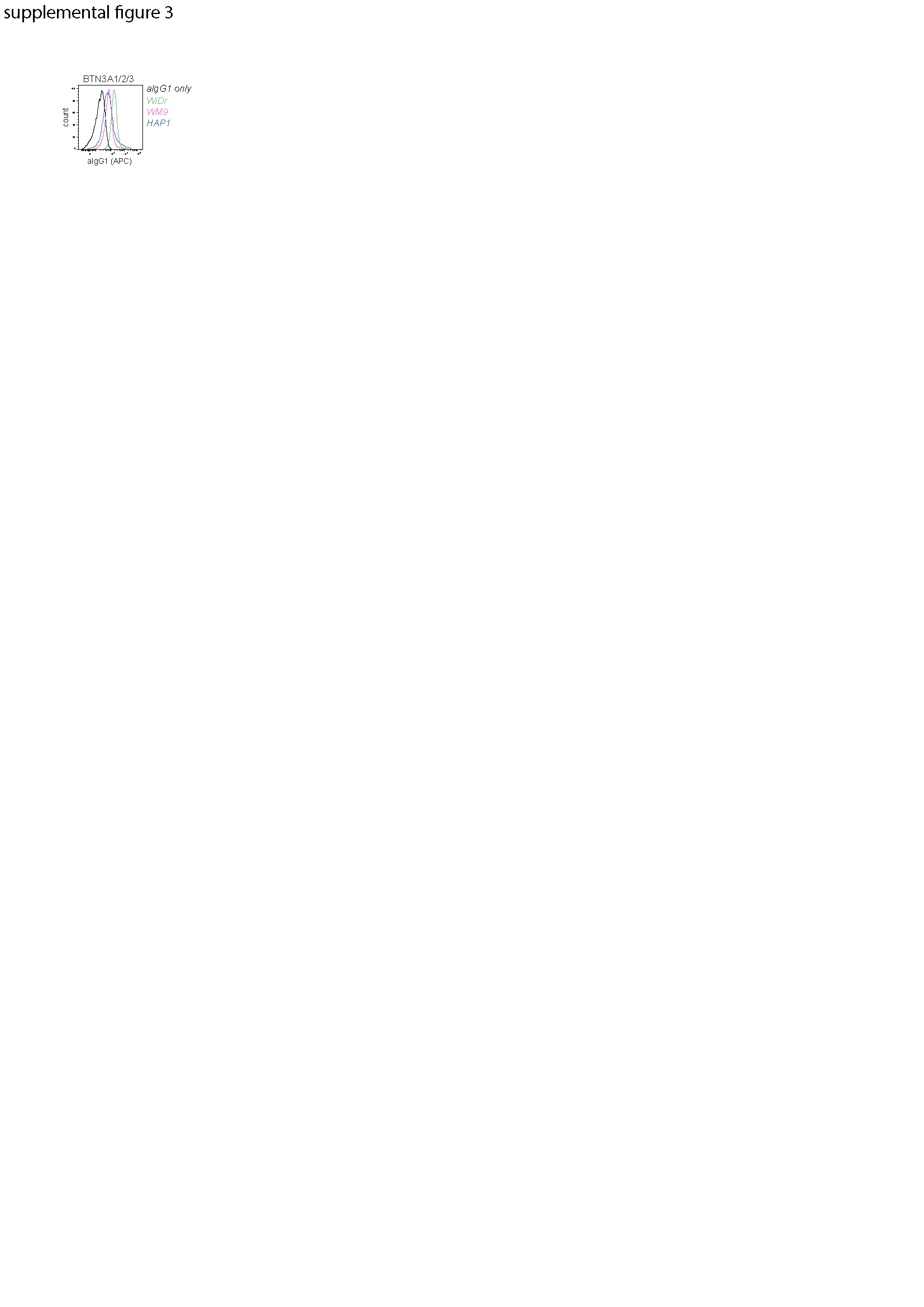

Supplement: Supplementary Figure 3 — Cell surface expression profiles of BTN3A1/2/3 by WiDr, WM9 and HAP1 cell lines. Represented is a flow cytometry plot of WiDr (green), WM9 (pink) and HAP1 (blue) cells incubated with anti-BTN3A1/2/3 and anti-IgG1 (APC). Included is a background control of anti-IgG1 only on WiDr cells (black) which is representative for the background on WM9 and HAP1 cells. [file Image_3.tiff]
